# TGF‐β/Smad2 signalling regulates enchondral bone formation of Gli1^+^ periosteal cells during fracture healing

**DOI:** 10.1111/cpr.12904

**Published:** 2020-09-30

**Authors:** Chenjie Xia, Qinwen Ge, Liang Fang, Huan Yu, Zhen Zou, Peng Zhang, Shuaijie Lv, Peijian Tong, Luwei Xiao, Di Chen, Ping‐er Wang, Hongting Jin

**Affiliations:** ^1^ Institute of Orthopadics and Traumatology The First Affiliated Hospital of Zhejiang Chinese Medical University Hangzhou China; ^2^ The First College of Clinical Medicine Zhejiang Chinese Medical University Hangzhou China; ^3^ Department of Orthopedic Surgery Ningbo Medical Center Lihuili Hospital Ningbo China; ^4^ Department of Orthopedic Surgery The First Affiliated Hospital of Zhejiang Chinese Medical University Hangzhou China; ^5^ Research Center for Human Tissues and Organs Degeneration Shenzhen Institutes of Advanced Technology Chinese Academy of Sciences Shenzhen China

**Keywords:** enchondral bone formation, fracture healing, Gli1, periosteum, TGF‐β signalling

## Abstract

**Objectives:**

Most bone fracture heals through enchondral bone formation that relies on the involvement of periosteal progenitor cells. However, the identity of periosteal progenitor cells and the regulatory mechanism of their proliferation and differentiation remain unclear. The aim of this study was to investigate whether *Gli1‐Cre^ERT2^* can identify a population of murine periosteal progenitor cells and the role of TGF‐β signalling in periosteal progenitor cells on fracture healing.

**Materials and methods:**

Double heterozygous *Gli1‐CreER^T2^;Rosa26‐tdTomato^flox/wt^* mice were sacrificed at different time points for tracing the fate of Gli1^+^ cells in both intact and fracture bone. *Gli1‐CreER^T2^*‐mediated *Tgfbr2* knockout (*Gli1‐CreER^T2^;Tgfbr2^flox/flox^*) mice were subjected to fracture surgery. At 4, 7, 10, 14 and 21 days post‐surgery, tibia samples were harvested for tissue analyses including μCT, histology, real‐time PCR and immunofluorescence staining.

**Results:**

Through cell lineage‐tracing experiments, we have revealed that *Gli1‐Cre^ER^^T2^* can be used to identify a subpopulation of periosteal progenitor cells in vivo that persistently reside in periosteum and contribute to osteochondral elements during fracture repair. During the healing process, TGF‐β signalling is continually activated in the reparative Gli1^+^ periosteal cells. Conditional knockout of *Tgfbr2* in these cells leads to a delayed and impaired enchondral bone formation, at least partially due to the reduced proliferation and chondrogenic and osteogenic differentiation of Gli1^+^ periosteal cells.

**Conclusions:**

TGF‐β signalling plays an essential role on fracture repair via regulating enchondral bone formation process of Gli1^+^ periosteal cells.

## Graphical abstract

Through cell lineage‐tracing experiments, we have revealed that *Gli1‐Cre^ERT2^* can be used to identify a subpopulation of periosteal progenitor cells in vivo that persistently reside in periosteum and contribute to osteochondral elements during fracture repair. TGF‐β signalling is continually activated in the reparative Gli1^+^ periosteal cells and regulates their proliferation and chondrogenic and osteogenic differentiation, which is essential for normal fracture healing.

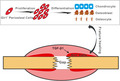

## 1 INTRODUCTION

Bone has a high regenerative capacity that enables most fractures healed in a native form and function.^1^ This reparative nature of bone relies mainly on the existence of local active progenitor cells.^2^, ^3^ Fracture healing is a complex process that undergoes three major biologically distinct but overlapping phases including haematoma, fracture callus formation and bone remodeling.^4^ Progenitor cells make differential contributions to each phase, such as recruitment and proliferation at the initial haematoma phase and chondrogenic and osteogenic differentiation at subsequent phases.^5^ Although the importance of progenitor cells to fracture healing have been well documented, the identity and regulatory mechanism of progenitor cells are still largely unknown.

Several potential sources of skeletal progenitor cells are proposed for bone regeneration, including bone marrow,^6^ periosteum,^7^ endosteum,^8^ adjacent soft tissue^9^, ^10^ and vascular walls.^11^ Recent findings highlight the importance of progenitor cells within periosteum since they can give rise directly to cartilage and bone during the healing process.^1^, ^5^, ^12^ Removal of the periosteum tissue leads to clinical delayed union or nonunion of fractures with no fracture callus formation.^13^ Over the last decade, with development of lineage‐tracing technology, some periosteal markers, such as Prx1,^14^ Sox9,^15^ aSMA^16^ and CTSK^17^ have been identified in mice. Nevertheless, it still needs to vigorously investigate the promising progenitor cell populations for better defining the contribution of periosteal progenitor cells to fracture healing. Gli1 is a mediator of Hedgehog signalling that controls bone development.^18^ Previous studies have revealed that Gli1^+^ cells within the craniofacial sutures^19^ and growth plate^20^, ^21^ have the progenitor properties, and more remarkably, they largely contribute to fracture callus^20^ and heterotopic bone formation.^22^ Here, we seek to further determine whether Gli1 can identify a population of periosteal progenitor cells during fracture healing.

Amongst numerous growth factors and cytokines, transforming growth factor β (TGF‐β) is one of the most important factors in regulation of fracture healing.^23^, ^24^ Clinical evidence shows a rapid elevation of TGF‐β serum responding to fracture in patients.^25^ Patients with low TGF‐β level are tending to have delayed union or nonunion.^26^, ^27^ TGF‐β regulates bone regeneration mainly via the Smad‐dependent canonical pathway.^28^ After TGF‐β ligand binding to type II receptor (TGF‐βRII), phosphorylated Smad2 in turn is translocated into the nucleus and activates the downstream target genes which are responsible for cell proliferation, cell differentiation and extracellular matrix production.^24^, ^29^ Currently, the role of TGF‐β/Smad2 signalling in periosteal progenitor cells remains unclear in the context of fracture repair.

In the present study, we hypothesize that TGF‐β/Smad2 signalling can regulate the reparative response of Gli1^+^ periosteal cells for murine fracture healing. By tracing the fate of Gli1‐expressing lineage cells in both intact and fracture tibiae in mice, we have demonstrated that Gli1 identifies a population of periosteal cells in vivo that persistently resides in periosteum tissue and also can give rise to chondrocytes and osteoblasts during fracture healing process. Furthermore, by utilizing *Gli1‐Cre*‐mediated *Tgfbr2* inducible knockout mice, we have revealed that inhibition of TGF‐β/Smad2 signalling in Gli1^+^ periosteal cells negatively affects their proliferation, chondrogenic and osteogenic differentiation, therefore resulting in an impaired endochondral bone formation in fracture healing.

## 2 MATERIALS AND METHODS

### 2.1 Animals

*Gli1‐CreER^T2^* mice, *Rosa26‐tdTomato^flox/flox^* mice and *Tgfbr2^flox/flox^* mice were obtained from Jackson Laboratory. For lineage‐tracing experiments, a double heterozygous *Gli1‐CreER^T2^;Rosa26‐tdTomato^flox/wt^* (*Tomato^Gli1ER^*) mice were generated, and tamoxifen (1 mg/10 g body weight/day, diluted in corn oil) was injected intraperitoneally into 1‐month‐old mice for 3 consecutive days. To investigate the role of TGF‐β signalling in Gli1^+^ periosteal cells in fracture healing, *Gli1‐CreER^T2^;Tgfbr2^flox/flox^* (*Tgfbr2^Gli1ER^*) mice and *Gli1‐CreER^T2^;Tgfbr2^flox/flox^; Rosa26‐tdTomato^flox/wt^* (*Tgfbr2^Gli1ER^;ROSA^tdTomato^*) mice were generated following with 3 consecutive intraperitoneal injections of tamoxifen at 1 month of age, or mice were subcutaneously injected with TGF‐β neutralizing antibody (5 mg/kg body weight) at the fracture site once every 2 days starting immediately after fracture. The specific information of transgenic mice were provide in Table 1. Both male and female mice were used in lineage‐tracing studies, but only males were subjected to fracture surgery to avoid sex‐dependent difference. All animal experiments were approved by the Animal Ethics Committee of Zhejiang Chinese Medical University (LZ12H27001).

**Table 1:** Information of transgenetic mice

Transgenetic mice	Abbreviation	Sources
*Gli1‐CreER^T2^*	—	Jackson Lab
*Rosa26‐tdTomato^flox/flox^*	—	Jackson Lab
*Tgfbr2^flox/flox^*	—	Jackson Lab
*Gli1‐CreER^T2^;Rosa26‐tdTomato^flox/wt^*	*Tomato^Gli1ER^*	Breeding
*Gli1‐CreER^T2^;Tgfbr2^flox/flox^*	*Tgfbr2^Gli1ER^*	Breeding
*Gli1‐CreER^T2^;Tgfbr2^flox/flox^;Rosa26‐tdTomato^flox/wt^*	*Tgfbr2^Gli1ER^;ROSA^tdTomato^*	Breeding

### 2.2 Tibial fracture model

An open transverse tibial fracture model was established unilaterally in the male mice as previously described.^30^, ^31^ Briefly, an incision of 1 cm was made along the surface of tibial crest after mice were anesthetized by intraperitoneal injection of pentobarbital (60 mg/kg body weight). Medial to the patellar tendon, a 26‐gauge needle was inserted into the tibial intramedullary cavity through the tibial platform. The needle was removed followed by a transverse cut with a NO.11 surgical blade at the midpoint of the tibia. The transverse fracture was then fixed again by the needle. Mice were sacrificed at 4, 7, 10, 14 and 35 days post‐fracture, and tibia samples were harvested for further analysis.

To determine the importance of periosteum to bone repair, we removed 0.1 mm periosteum tissue on the fractured tibia. Briefly, after the transverse fracture, the antero‐ and posterior‐lateral periosteum was striped off by the NO.11 surgical blade. Mice were sacrificed at 4 and 14 days post‐fracture for phenotypical analyses.

### 2.3 CidU administration

*Tomato^Gli1ER^* mice received the artificial nucleoside chlorodeoxyuridine (CidU; Sigma; St. Louis, USA) immediately after fracture surgery via subcutaneous injection once at a concentration of 10 mg/mL followed by oral administration for another 3 days at a concentration of 1 mg/mL.^32^ Tibia samples were harvested next day for in vivo cell proliferation analysis.

### 2.4 μCT analysis

Fractured tibia samples were scanned with a micro‐computed tomography (μCT) (Skyscan1176, Belgium) at a resolution of 10 μm. Three‐dimensional (3D) structure of facture callus was reconstructed using nrecon software. Morphometric analysis including cortical bone volume (CBV, mm^3^), cortical bone surface/cortical bone volume (CBS/CBV, 1/mm), callus total volume (TV, mm^3^), callus bone volume (BV, mm^3^) and callus mineralized volume fraction (BV/TV, %) was measured as previously described.^30^, ^31^

### 2.5 Histology and histomorphometry

Tibia samples were processed for 3‐μm‐thick paraffin section or 10‐μm‐thick frozen section. The sections were stained with DAPI staining for cell lineage‐tracing or Alcian Blue Hematoxylin (ABH)/Orange G for histological analysis.^33^ The total periosteal callus area, the cartilaginous callus area and the mineralized bone callus area were measured using OsteoMetrics software (Decatur, GA). Furthermore, the cartilage area of the periosteal callus area (Cg.Ar/Ps.Cl.Ar, %) and the mineralized bone area of the periosteal callus area (Md.Ar/Ps.Cl.Ar, %) were calculated to respectively evaluate the cartilage and mineralized bone formation, as previously described.^30^, ^31^ Abbreviations: Ar, area; Cg, cartilage; Ps, periosteal; Md, mineralized; and Cl, callus.

### 2.6 Immunofluorescence assay

Immunofluorescence (IF) assay were performed on the frozen sections according to the previously established procedures.^33^ Briefly, sections were treated with pepsinum (ZSGB Biotechnology, Beijing, China) at 37°C for 15 minutes or 0.01 mol/L citrate buffer (Solarbio, Beijing, China) at 60°C for 4 hours. Next, sections were incubated in primary antibodies overnight at 4°C and the antibodies applied in this study included TGF‐β1 (diluted 1:200, Arigo Biolaboratories, Shanghai, China), Phospho‐Smad2 (p‐Smad2; diluted 1:200, Thermo Fisher Scientific, Pittsburgh, PA, USA), type II collagen (Col‐II; diluted 1:200, Abcam, Cambridge, UK), osteocalcin (OCN; diluted 1:200, Takara, UK) and CidU (diluted 1:100, Abcam, Cambridge, UK). After incubation with secondary antibodies for 20 minutes, tissue sections were counter‐stained with 4',6‐diamidino‐2‐phenylindole (DAPI). Fluorescent quantitative analysis was calculated from three mice (one representative section per mouse) using image‐pro plus software.

### 2.7 Quantitative gene expression analysis

Fracture callus including 1 mm adjacent bone tissue on either side of the fracture line were collected for real‐time PCR analysis as previously described.^30^, ^31^ Primer sequences for target genes are provided in Table 2.

**Table 2:** Primer name and sequences for PCR analysis

Primer Name	Forward	Reverse
GAPDH	5′‐ AGGTCGGTGTGAACGGATTTG ‐3′	5′‐TGTAGACCATGTAGTTGAGGTCA ‐3′
Col2a1	5′‐TGGTCCTCTGGGCATCTCAGGC‐3′	5′‐GGTGAACCTGCTGTTGCCCTCA‐3′
Col10a1	5′‐ACCCCAAGGACCTAAAGGAA‐3′	5′‐CCCCAGGATACCCTGTTTTT‐3′
Runx2	5′‐GAGGGCACAAGTTCTATCTGGA‐3′	5′‐GGTGGTCCGCGATGATCTC‐3′
Osteocalcin	5′‐AGGGAGGATCAAGTCCCG ‐3′	5′‐GAACAGACTCCGGCGCTA‐3′
Tgfbr2	5′‐AGATGGCTCGCTGAACACTACCAA‐3′	5′‐AGAATCCTGCTGCCTCTGGTCTTT‐3′

### 2.8 Statistical analysis

Statistical analyses including one‐way ANOVA followed by Tukey's test and unpaired Student's *t* tests were performed with the software of spss 20.0. ^*^P < .05 was considered statistically significant.

## 3 RESULTS

### 3.1 Postnatal Gli1^+^ cells persistently reside in periosteum and contribute to fracture callus formation

**Figure 1:**
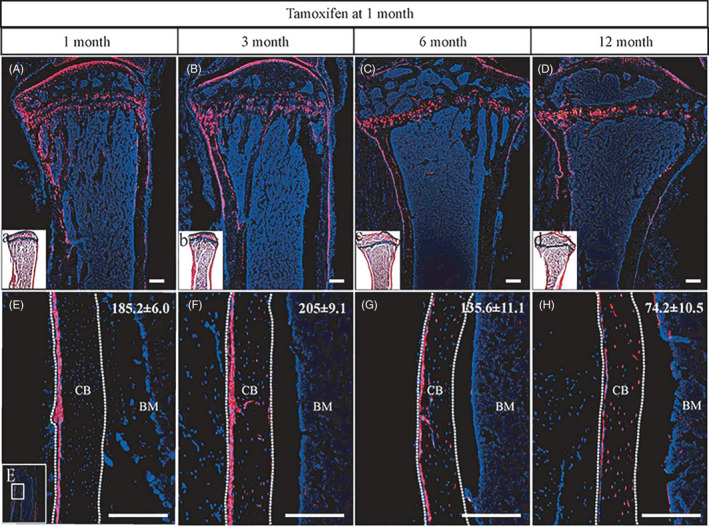
Postnatal Gli1^+^ cells reside in periosteum. *Tomato^Gli1ER^* mice were administered with tamoxifen at 1 mo of age and analysed at 1, 3, 6 and 12 mo later. A‐D, Representative images of tibiae from each time point mapped Gli1^+^ cells evidently in articular cartilage, growth plate, chondro‐osseous junction and periosteum. (a‐d) ABH stained images of tibiae were an adjacent section to (A‐D), respectively, and (a‐d) indicated the expression locations of Gli1^+^ cells at each time point in (A‐D). E‐H, The numbers of Gli1^+^ cells in periosteal area of the diaphyseal cortical bones. CB: cortical bone, BM: bone marrow. Red: tdTomato^+^ cells, blue: nuclear staining by DAPI. Scale bars: 1000 µm

To investigate the contribution of Gli1^+^ cells during the skeleton development, *Tomato^Gli1ER^* mice were given 3 doses of tamoxifen via intraperitoneal injection at 1 month of age, by which Gli1^+^ cells and their descendants permanently expressed red fluorescent protein tdTomato. Analyses of the intact tibiae at 1, 3, 6 and 12 months after the last dose identified Gli1^+^ cells evidently in the following domains: articular cartilage, growth plate, chondro‐osseous junction and periosteum (Figure 1A‐D and 1a‐d). As the fate of Gli1^+^ cells in other locations have been well clarified previously,^20^, ^21^ we chose to focus our study on the Gli1^+^ cells resident within periosteum. During 1 year of chase, Gli1^+^ periosteal cells and their descendants were found to persistently exist in the periosteum, which was more prevalent in the proximal than that in the medial (Figure 1A‐D). The number of Gli1^+^ periosteal cell population peaked at 3 months after tamoxifen induction and then gradually reduced at 6 and 12 months after tamoxifen induction. By 12 month, progenitor cells derived osteoblasts and osteocytes were observed expressing red fluorescence within the cortical bone (Figure 1E‐H), suggesting the differentiation capacity of Gli1^+^ periosteal cells.

**Figure 2:**
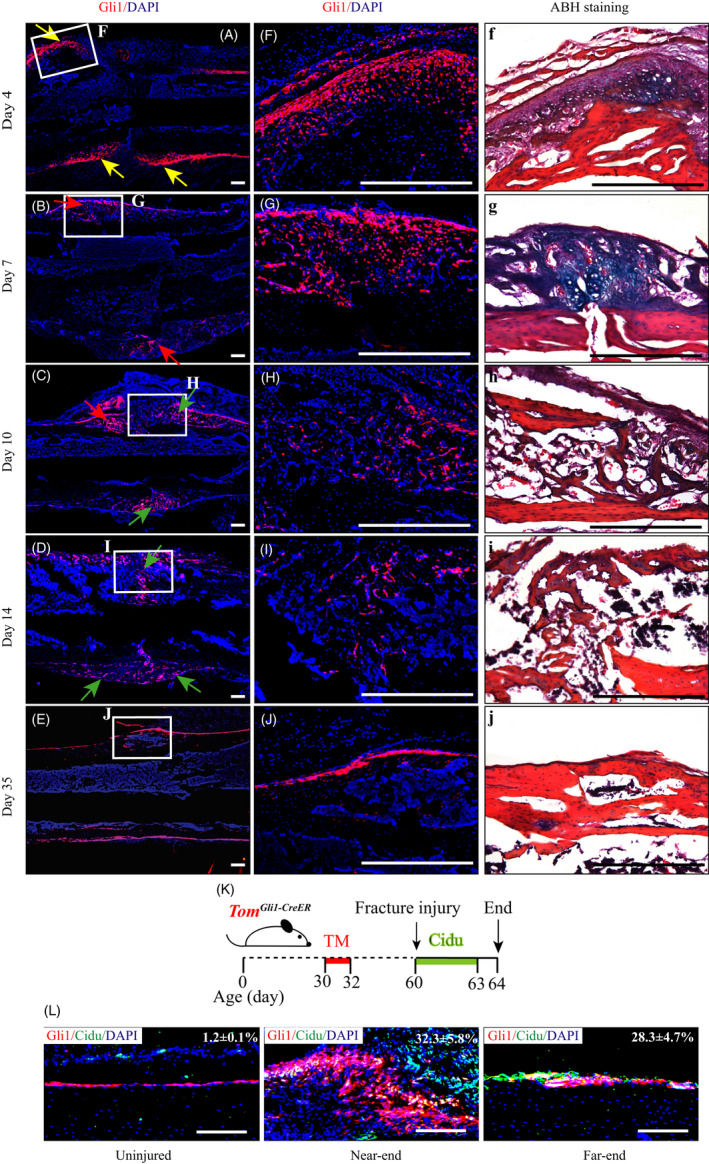
Gli1^+^ periosteal cells undergo proliferation and differentiation into chondrocytes, osteoblasts and osteocytes during fracture healing. *Tomato^Gli1ER^* mice induced with tamoxifen at 1 mo of age were subjected to the fracture surgery at 10 wk of age and sacrificed 4, 7, 10, 14 and 35 d later. A‐E, Representative immunofluorescence images of fractured tibiae from each time point. F‐J, High magnification images of local fracture sites in (A‐E), respectively. (f‐j) ABH stained images were an adjacent section to (F‐J) respectively, indicating the components that Gli1^+^ cells proliferated and differentiated at each time point. (A, F, f) Gli1^+^ cells largely expanded on the periosteal surface closed to the fracture site at day 4. Yellow arrows: expanded periosteum. B‐D, G‐I, g‐i, Gli1^+^ cells differentiated into chondrocytes, osteoblasts and osteocytes to form fracture callus at days 7‐14. Red arrows: chondrogenic differentiated Gli1^+^ cells. Green arrows: osteogenic differentiated Gli1^+^ cells. (E, J, j) Gli1^+^ cells were presented in the newly formed periosteum at day 35. K, Schematic experimental design for data in (L). L, Gli1^+^ periosteal cells both near to and away from the fracture sites highly expressed immunofluorescence signal of Cidu (green) at day 4 post‐fracture. Red: tdTomato^+^ cells, blue: nuclear staining by DAPI. Scale bars: 1000 µm

To investigate the role of Gli1^+^ periosteal cells in bone regeneration, *Tomato^Gli1ER^* mice induced with tamoxifen at 1 month of age were subjected to tibia fracture surgery at 10‐week‐old of age. Histological analyses from fluorescent images and ABH staining showed that Gli1^+^ periosteal cells extensively expanded after 4 days post‐fracture (Figure 2A,F, 2f, yellow arrows), gradually migrated to the fracture ends and meanwhile differentiated into chondrocytes (Figure 2B‐D, G,I, 2g‐i, red arrows), osteoblasts and osteocytes (Figure 2B‐D, G‐I, 2g‐i, green arrows) by days 7‐14 post‐fracture, and returned to the periosteum of reconstructed bone at day 35 (Figure 2E,J, 2j). Furthermore, *Tomato^Gli1ER^* mice were administrated with CidU for 4 consecutive days to detect the proliferation of Gli1^+^ periosteal cells (Figure 2K). Consistent with the histological observation of expanded periosteal tissue at the initial stage of fracture, Gli1^+^ periosteal cells both near to and far away from the fracture site largely expressed green fluorescence of CidU at day 4 post‐fracture (Figure 2L), indicating that Gli1^+^ periosteal cells underwent a rapid proliferation.

**Figure 3:**
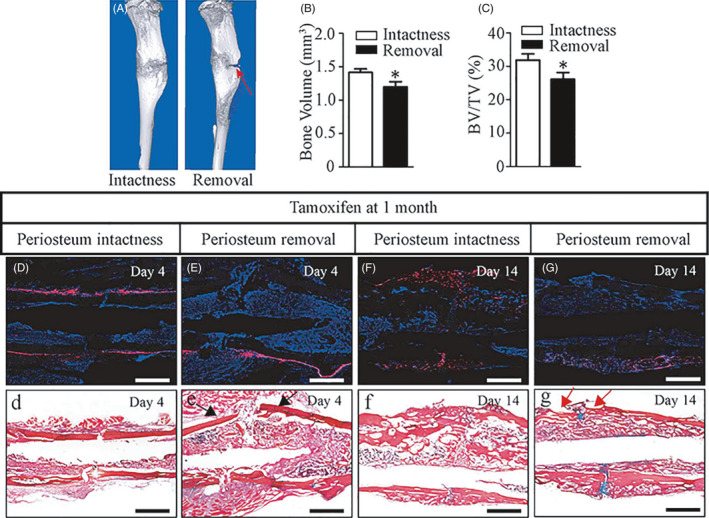
Periosteal‐derived Gli1^+^ cells are essential for fracture healing. *Tomato^Gli1ER^* mice induced with tamoxifen at 1 mo of age were subjected to tibia fracture surgery combined with removing antero‐ and posterior‐lateral periosteum at 10‐wk‐old. A, Representative three‐dimensional (3D) µCT images showed a distinct fracture line (red arrow) at day 14 in the periosteum removed mice. B, C, Quantitative μCT analysis of bone volume and BV/TV for fracture callus tissues at day 14. D‐G, No Gli1^+^ cells were presented in the periosteum removed side at days 4 and 14 post‐fracture. (d‐g) ABH staining of an adjacent section to (D‐G), respectively. The periosteum removed mice presented a deficiency of periosteal expansion at day 4 (black arrows) and almost no bone callus formation (red arrows) at day 14

To further demonstrate the importance of Gli1^+^ periosteal cells to fracture healing, we performed an extra surgery to remove the antero‐ and posterior‐lateral periosteum on the fractured tibia of *Tomato^Gli1ER^* mice. 3D images showed a nonunified fracture line in the periosteum removal mice compared to the periosteum intact ones at day 14 post‐fracture (Figure 3A, red arrow). Quantitative μCT analysis indicated that both BV and BV/TV of fracture callus were significantly decreased in the periosteum removal mice (Figure 3B,C). Fluorescent staining revealed that almost no Gli1^+^ periosteal cells were appeared at days 4 and 14 post‐fracture in the periosteum removal fractures (Figure 3D‐G). As a result, the periosteal tissue expansion (Figure 3D,E, black arrows) and fracture callus formation were remarkedly decreased (Figure 3F, 3g, red arrows). Altogether, these findings indicated that Gli1^+^ periosteal cells were essential to normal fracture healing, and Gli1^+^ cells residing in other locations could not be recruited to repair fracture.

### 3.2 Continuous activation of TGF‐β/Smad2 signalling in Gli1^+^ periosteal cells during fracture healing

**Figure 4:**
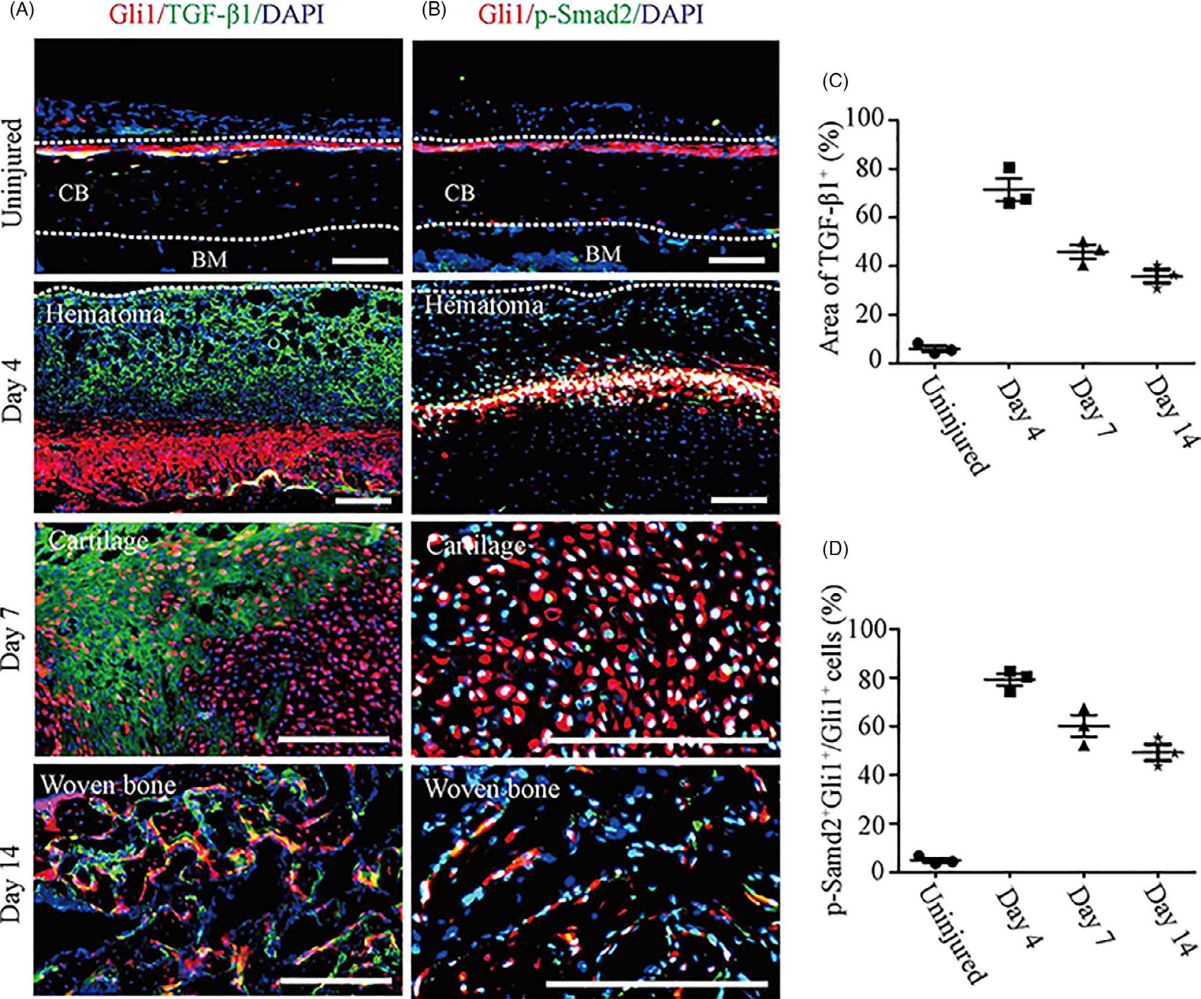
TGF‐β/Smad2 signalling is activated in Gli1^+^ periosteal cells during fracture healing. A, Immunofluorescence signal of TGF‐β1 (green) in the uninjured cortical bone, fracture haematoma at day 4, cartilage matrix at day 7 and bone matrix at day 14. B, Immunofluorescence signal of p‐Smad2 (green) in the uninjured Gli1^+^ periosteal cells, the expending Gli1^+^ periosteal cells at day 4, the chondrogenic differentiated Gli1^+^ cells at day 7 and the osteogenic differentiated Gli1^+^ cells at day 14. C, Percentage of TGF‐β1^+^ area quantified in the respective regions at different time points. D, Percentage of p‐Smad2^+^ tdTomato^+^ over tdTomato^+^ cells quantified in the respective regions at different time points. CB: cortical bone, BM: bone marrow, Red: tdTomato^+^ cells, blue: nuclear staining by DAPI. Scale bars: 1000 µm

To evaluate the expression of TGF‐β/Smad2 signalling in Gli1^+^ periosteal cells during fracture healing, IF assay was performed in the intact and fractured tibiae by using TGF‐β1 and p‐Smad2 antibodies (Figure 4A‐D). At day 4 post‐fracture, TGF‐β1 expression was highly increased in the fracture haematoma tissue compared to the uninjured tibiae. Importantly, almost all Gli1^+^ periosteal cells co‐expressed the green fluorescence of p‐Smad2. At day 7 post‐fracture, TGF‐β1 was largely accumulated in the cartilaginous template, and more than 50% of Gli1^+^ cells was found to differentiate into chondrocytes which were also p‐Smad2^+^. At day 14 post‐fracture, TGF‐β1 expression persisted in the woven bone and about half of Gli1^+^;p‐Smad2^+^ over total Gli1^+^ periosteal cells differentiated into osteoblasts. These findings indicated that TGF‐β/Smad2 signalling was continuously activated in Gli1^+^ periosteal cells throughout the healing process and may govern the differentiation of periosteal cells.

### 3.3 Local application of TGF‐β1 neutralizing antibody results in a delayed and impaired endochondral bone formation in fractured mice

**Figure 5:**
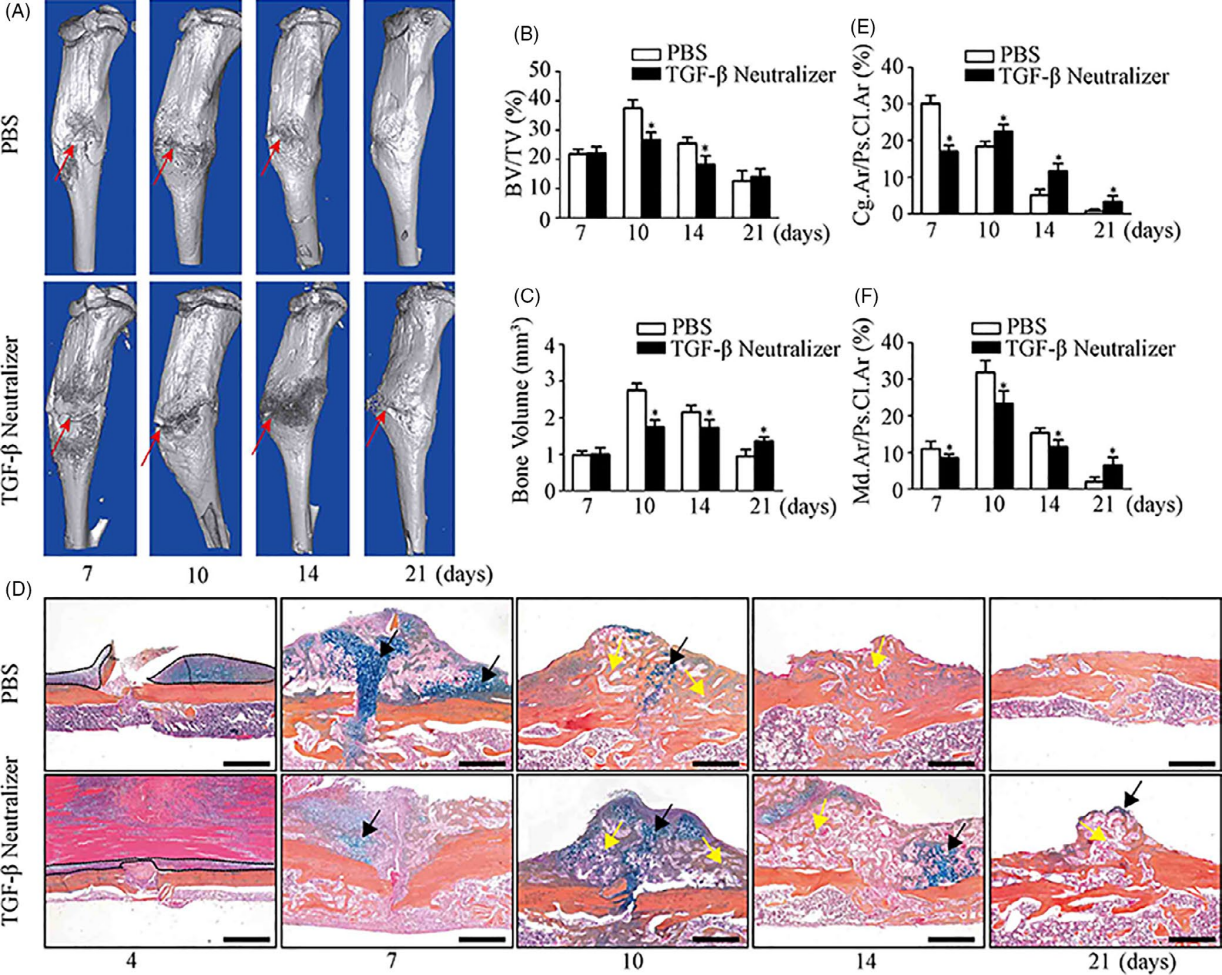
TGF‐β neutralizing antibody results in a delayed enchondral bone formation in fractured mice. TGF‐β neutralizing antibody (5 mg/kg body, once every 2 d) was subcutaneously injected into fractured regions immediately after fracture. A, Representative µCT images showed a distinct fracture line at day 21 (red arrow) in TGF‐β neutralizing antibody administrated mice. B, C, Quantitative μCT analysis of callus bone volume (BV) and callus mineralized volume fraction (BV/TV) at different time points. D, ABH staining of fracture callus at different time points. Dotted lines: the expanding periosteum, black arrows: cartilage area, yellow arrow: woven bone area. E, Percentage of cartilage area over periosteal callus area (Cg.Ar/Ps.Cl.Ar, %). F, Percentage of woven bone area over periosteal callus area (Md.Ar/Ps.Cl.Ar, %). Scale bars: 1000 µm

We analysed the essential role of TGF‐β1 in fractured microenvironment to fracture healing through subcutaneously injecting TGF‐β neutralizing antibody at the fracture site. μCT analysis showed a clear fracture line at day 21 post‐fracture (Figure 5A) and a significant decrease of BV and BV/TV in fracture callus at days 10 and 14 (Figure 5B,C) in the TGF‐β1 neutralizing antibody treated mice. ABH staining further revealed a reduction of periosteal expansion at day 4 (Figure 5D, Black dotted line), a weak and delayed cartilage formation at days 7 and 10 (Figure 5D, Black arrow), a significant deceased woven bone formation at days 10 and 14 (Figure 5D, Yellow arrow), and massive unabsorbed cartilage and woven bone at day 21 post‐fracture (Figure 5D, Black arrow and Yellow arrow, respectively) in mice with local application of TGF‐β1 neutralizing antibody compare to the PBS treated mice. Consistently, histomorphometric quantification of Cg.Ar/Ps.Cl.Ar and Md.Ar/Ps.Cl.Ar confirmed the significant decrease of cartilage area at day 7 (Figure 5E) and the largely decreased mineralized bone area at days 7, 10 and 14 post‐fracture (Figure 5F) in the periosteal callus area of TGF‐β1 neutralizing antibody treated mice. These data indicated that local application of TGF‐β1 neutralizing antibody would lead to a delayed and impaired endochondral bone formation in fractured mice.

### 3.4 Deletion of *Tgfbr2* in Gli1^+^ periosteal cells leads to a delayed endochondral bone formation in fractured mice

**Figure 6:**
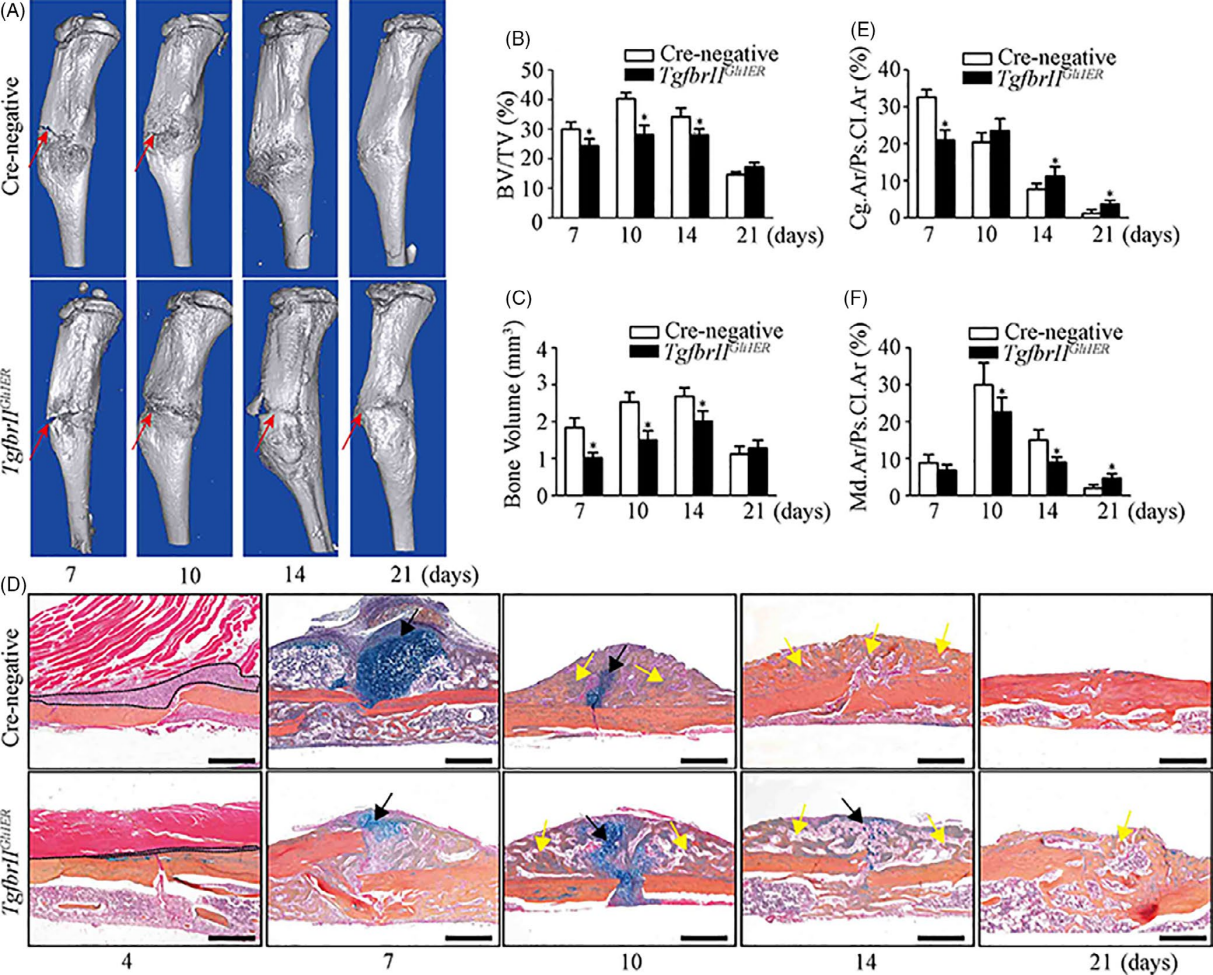
Deletion of *Tgfbr2* in Gli1^+^ periosteal cells leads to a delayed and impaired enchondral bone formation in fractured mice. *Tgfbr2^Gli1ER^* mice induced with tamoxifen at 1 mo of age were subjected to tibial fracture surgery at 10 wk of age. A, Representative µCT images showed the distinct fracture lines at day 14 and 21 (red arrow) in *Tgfbr2^Gli1ER^* mice compared to Cre‐negative mice. B, C, Quantitative μCT analysis of callus bone volume (BV) and callus mineralized volume fraction (BV/TV) at different time points. D, ABH staining of fracture callus at different time points. Dotted lines: the expanding periosteum, black arrows: cartilage area, yellow arrow: woven bone area. E, Percentage of cartilage area over periosteal callus area (Cg.Ar/Ps.Cl.Ar, %). F, Percentage of woven bone area over periosteal callus area (Md.Ar/Ps.Cl.Ar, %). Scale bars: 1000 µm

In order to determine the effects of TGF‐β/Smad2 signalling on regulating the differentiation of Gli1^+^ periosteal cells in the healing process, *Gli1‐Cre*‐mediated *Tgfbr2* conditional knockout (*Tgfbr2^Gli1ER^*) mice were used and *Tgfbr2* gene deletion was achieved by administering 3 consecutive doses of tamoxifen at 1 month of age. Firstly, we analysed the morphologic changes of tibiae in 10‐week‐old *Tgfbr2^Gli1ER^* mice by histology and μCT. Compared to the control mice, *Tgfbr2^Gli1ER^* mice appeared to be morphologically normal and exhibited no difference in growth plate, cortical bone and trabecular bone of tibiae (Figure S1A,B). Consistent with histology, μCT further confirmed no difference in the parameters analysed from tibiae of cortical bone including CBV and CBS/CBV (Figure S1C,D). Then, *Tgfbr2^Gli1ER^* mice were subjected to fracture surgery. Representative μCT images showed a delayed fracture repair in *Tgfbr2^Gli1ER^* mice with the evidence of the unclosed fracture lines at days 14 and 21 post‐fracture compared to the control mice (Figure 6A, red arrows). Quantitative analysis showed that both BV and BV/TV of fracture callus were significantly decreased in *Tgfbr2^Gli1ER^* mice at days 7, 10 and 14 post‐fracture (Figure 6B,C).

Histological analyses also revealed a delayed and impaired endochondral bone formation in *Tgfbr2^Gli1ER^* mice. Compared to the control mice, *Tgfbr2^Gli1ER^* mice presented a reduced periosteal expansion at day 4 post‐fracture (Figure 6D, dotted lines). At day 7 post‐fracture, diminished cartilage tissue (Figure 6D, Black arrow) were formed in *Tgfbr2^Gli1ER^* mice, compared to that in the control mice. At day 10 post‐fracture, *Tgfbr2^Gli1ER^* mice had a significant increase of cartilage (Figure 6D, Black arrow), while most of cartilage in the control mice were already replaced by woven bone (Figure 6D, Yellow arrow). By day 14 post‐fracture, cartilage was replaced by woven bone in the control mice. However, cartilage remnant was still observed in *Tgfbr2^Gli1ER^* mice (Figure 6D, Black arrow) with reduced woven bone tissue (Figure 6D, Yellow arrow). At day 21 post‐fracture, we could still observe unabsorbed woven bone in *Tgfbr2^Gli1ER^* mice (Figure 6D, Yellow arrow). Consistently, histomorphometric quantification of Cg.Ar/Ps.Cl.Ar and Md.Ar/Ps.Cl.Ar showed that the percentage of cartilage area in periosteal callus area was significantly decreased at day 7 post‐fracture, but largely increased at days 10, 14 and 21 in *Tgfbr2^Gli1ER^* mice compared to the control mice (Figure 6E); and the radio of mineralized bone area in periosteal callus area was significantly decreased at days 10 and 14 post‐fracture, but significantly increased at day 21 in *Tgfbr2^Gli1ER^* mice compared to the control mice (Figure 6F). These data suggested a reduction of chondrogenic and osteogenic differentiation in *Tgfbr2^Gli1ER^* fractures.

**Figure 7:**
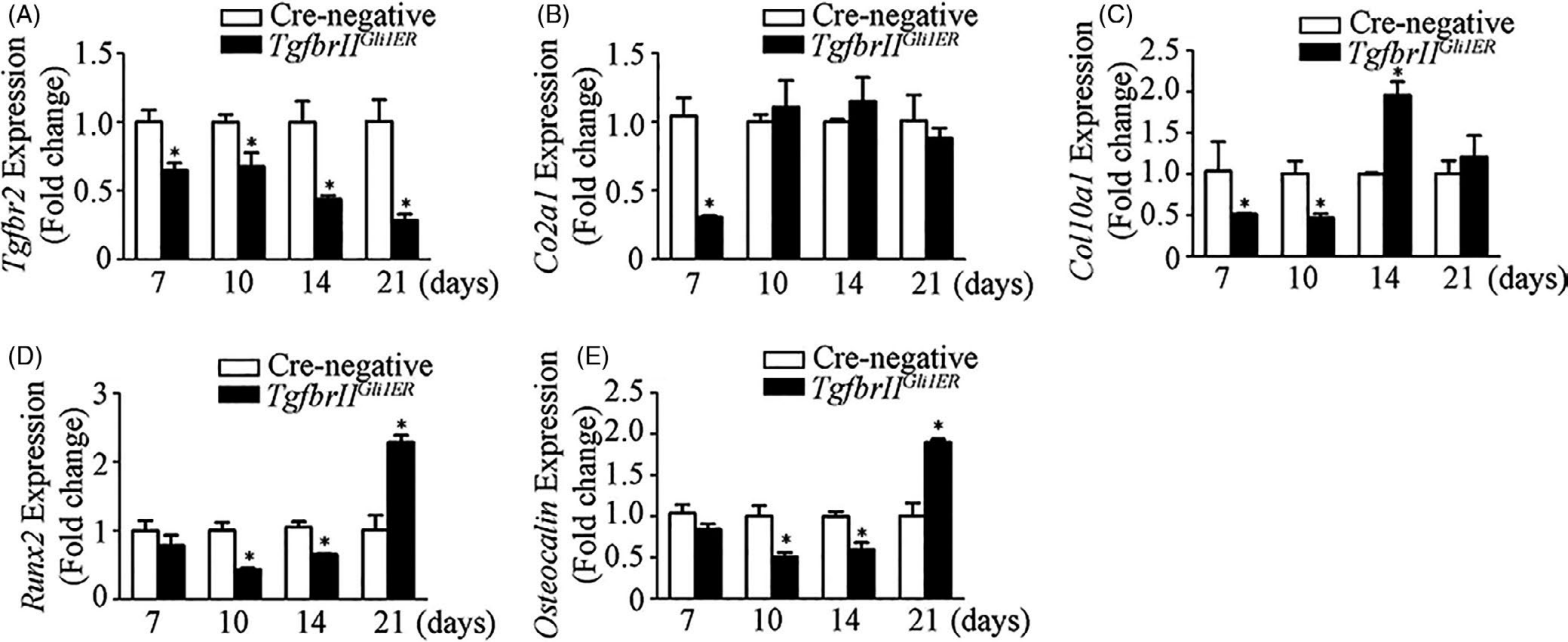
Deletion of *Tgfbr2* in Gli1^+^ periosteal cells down‐regulates expressions of chondrocyte‐ and osteoblast‐specific marker genes in callus tissues. Total RNA was extracted from callus tissues (n = 3) of *Tgfbr2^Gli1ER^* mice at different time points. A, Expression of *Tgfbr2* was decreased at day 7‐21. B, Expression of *Col2a1* was decreased at day 7. C, Expression of *Col10a1* was decreased at day 7 and 10, but increased at day 14. D, E, Expression of *Runx2* and *osteocalin* was decreased at day 10 and 14, but increased at day 21

The changes of endochondral bone formation were further confirmed by the expressions of cartilage‐ and bone‐related genes. Compared to the control mice, *Tgfbr2* mRNA was remarkably decreased in *Tgfbr2^Gli1ER^* mice at different time points, indicating the continuous inhibition of TGF‐β/Smad2 signalling in the fracture callus (Figure 7A). *Tgfbr2^Gli1ER^* mice presented the significantly lower expressions of cartilage‐related genes (*Col2a1* and *Col10a1*) at day 7 post‐fracture (Figure 7B,C) and bone‐related genes (*Runx2* and *Osteocalcin*) at days 10 and 14 post‐fracture (Figure 7D,E). These data indicated *Tgfbr2* deficiency in Gli1^+^ periosteal cells led to a delayed and impaired endochondral bone formation in *Tgfbr2^Gli1ER^* mice, at least partially due to suppressed chondrogenic and osteogenic differentiation from Gli1^+^ periosteal cells.

### 3.5 Deletion of *Tgfbr2* in Gli1^+^ periosteal cells inhibits proliferation and differentiation of Gli1^+^ periosteal cells into chondrocytes and osteoblasts during healing process

**Figure 8:**
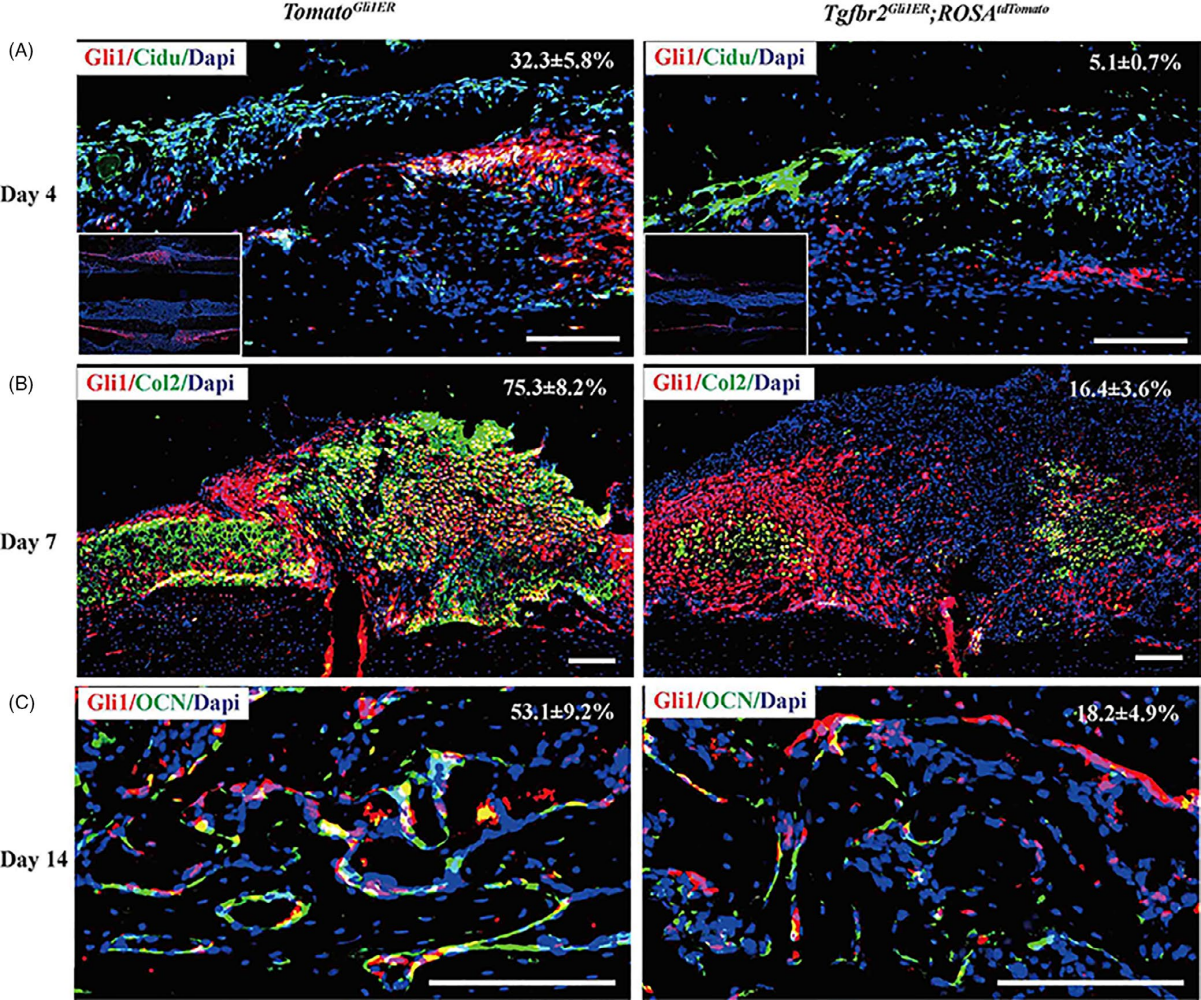
Deletion of *Tgfbr2* in Gli1^+^ periosteal cells inhibits proliferation and differentiation of Gli1^+^ periosteal cells into chondrocytes and osteoblasts during fracture healing. *Tgfbr2^Gli1ER^;ROSA^tdTomato^* mice induced with tamoxifen at 1 mo of age were subjected to fracture surgery at 10 wk of age. A, Percentage of tdTomato^+^; Cidu^+^ over tdTomato^+^ cells. B, Percentage of tdTomato^+^ chondrocytes surrounded by green fluorescence signal of Col‐II over tdTomato^+^ cells. C, Percentage of tdTomato^+^; Cidu^+^ over tdTomato^+^ cells. Scale bars: 1000 µm

We then examined the proliferation and differentiation of Gli1^+^ periosteal cells in *Tgfbr2^Gli1ER^* mice. *Gli1‐CreER^T2^;Tgfbr2^flox/flox^;Rosa26‐tdTomato^flox/wt^* (*Tgfbr2^Gli1ER^;ROSA^tdTomato^*) mice were generated to label Gli1^+^ cells with tdTomato red fluorescence and at the same time to delete *Tgfbr2* in Gli1^+^ cells. At day 4 after fracture, the percentage of Gli1^+^;CidU^+^ periosteal cells in *Tgfbr2^Gli1ER^;ROSA^tdTomato^* mice was much less than that in *Tomato^Gli1ER^* mice, indicating that the proliferative Gli1^+^ periosteal cells was reduced by loss of TGF‐β pathway (Figure 8A). Immunostaining analysis demonstrated that about 20% of Gli1^+^ periosteal cells differentiated into chondrocytes as shown by co‐staining with Col‐II green fluorescence in *Tgfbr2^Gli1ER^;ROSA^tdTomato^* mice at day 7 post‐fracture, while about 80% Gli1^+^ periosteal cells differentiated into chondrocytes in *Tomato^Gli1ER^* mice (Figure 8B). Similarly, less than 20% of OCN^+^ cells co‐expressed with tdTomato in *Tgfbr2^Gli1ER^;ROSA^tdTomato^* mice compared to about 50% in *Tomato^Gli1ER^* mice at day 14 post‐fracture (Figure 8C), indicating impaired osteoblast differentiation by loss of TGF‐β pathway. Altogether, these data indicated that inhibition of TGF‐β/Smad2 signalling in Gli1^+^ periosteal cells negatively affected their proliferation as well as chondrocyte and osteoblast differentiation during the process of endochondral bone formation.

## 4 DISCUSSIONS

Periosteum is the tissue that makes a major cellular contribution to both cartilage and bone formation during fracture healing process, and absence of periosteum leads to impaired fracture healing and even fracture nonunion.^1^, ^3^, ^5^, ^13^ The progenitor cells isolated from the periosteum show higher regenerative capacity compared to bone marrow mesenchymal stem cells and adipose‐derived mesenchymal cells; therefore, they are considered as ideal candidates for tissue engineering applications.^34^, ^35^ Previous studies have revealed that periosteum transplantation can successfully heal bone defects nonunion and in animal models.^36^, ^37^ However, the identify of the progenitor cells within periosteum is not well defined.

Different from the traditional in vitro cell behaviours identification, the in vivo lineage‐tracing experiments have enabled to identify periosteal progenitor cells in an unperturbed native environment.^38^ In the present study, by analysing *Tomato^Gli1ER^* lineage‐tracing transgenic mice, we have revealed a Gli1^+^ cell population persistently residing within the periosteum of long bone. Interestingly, Gli1^+^ periosteal cells and their descendants are abundant in juvenile mice but notably diminished by 7 months of age. Moreover, more Gli1^+^ osteoblasts and osteocytes within the cortical bone are observed in the aged mice, indicating that the Gli1^+^ periosteal cell population has self‐renew and differentiation capacity. Based on the critical contribution of periosteum to cortical bone modeling,^39^, ^40^ we speculate that these Gli1^+^ osteoblasts and osteocytes are more likely trans‐differentiated from the Gli1^+^ periosteal cells. In bone repair, fate mapping shows that Gli1^+^ periosteal cells proliferate and migrate towards the fracture ends at the early phase of healing, then differentiate into chondrocytes and osteoblasts and form fracture callus. Whether can Gli1^+^ cells residing within other locations of long bone migrate to participate in the healing, especially the growth plate‐derived Gli1^+^ cells which have shown the capability to continuously produce osteoblasts throughout life.^20^, ^21^ Our periosteum removal experiment demonstrated that no other Gli1^+^ cells are migrated and involved to supply the loss of Gli1^+^ periosteal cells caused by the removal of periosteum. Through tracing the fate of Gli1^+^ periosteal cells in intact and fracture tibiae, we have revealed Gli1^+^ periosteal cell as a progenitor cell due to its in vivo self‐renew and multipotency capability (producing chondrocytes and osteoblasts).^41^ Therefore, Gli1^+^ periosteal cells defines a subpopulation of progenitor cells contributing to the callus formation and fracture repair.

Endochondral bone formation is the way that most fractures heal,^42^ and Gli1^+^ periosteal cells substantially contribute to the osteochondral elements. IF analysis shows an abundant accumulation of TGF‐β1 in the fractured microenvironment that activates TGF‐β/Smad2 signalling in the Gli1^+^ periosteal cells during the initiation and progression of healing process. Inhibition of TGF‐β signalling in Gli1^+^ periosteal cells by local injection of TGF‐β neutralizing antibody or conditional deletion of *Tgfbr2* in Gli1^+^ periosteal cells, periosteal expansion and subsequent cartilage and bone callus formation are significantly reduced, leading to a delayed and impaired endochondral bone formation. Furthermore, although *Tgfbr2* deficiency in Gli1^+^ periosteal cells did not alter the long bone development process, the cell proliferation as well as chondrogenic and osteogenic differentiation are largely impaired in the context of fracture repair. These data confirmed that essential role of TGF‐β/Smad2 signalling in regulation of Gli1^+^ periosteal cell proliferation and differentiation as well as fracture healing.

It remains controversial regarding the effects of exogenous TGF‐β1 to bone fracture healing, tempering the potential usage of TGF‐β1 as a treatment. Previous studies have shown that local application of exogenous TGF‐β1 promotes fracture healing in animal models,^43^, ^44^ whereas some others obtains conflicting results.^45^ However, our data revealed that abundant endogenous TGF‐β1 is secreted in fractured microenvironment and is sufficient to trigger TGF‐β signalling regulating periosteal progenitor cell differentiation and endochondral bone formation, implicating exogenous TGF‐β1 as a potential treatment for fracture patients, especially for those with deficiency of TGF‐β pathway related molecules. It may be worth to evaluate dosage, administration route and cell specificity of TGF‐β1 as well as combination with other growth factors^24^ to determine whether TGF‐β1 can be used in clinic to treat fracture patients.

In summary, Gli1 can identify a population of periosteal progenitor cells in juvenile mice. TGF‐β/Smad2 signalling in Gli1^+^ periosteal cells is essential to the cell proliferation as well as chondrocyte and osteoblast differentiation in fracture healing.

## CONFLICT OF INTEREST

The authors declare no conflict of interest.

## AUTHOR CONTRIBUTIONS

Ping‐er Wang and Hongting Jin contributed to study conception and design. Chenjie Xia, Qinwen Ge, Liang Fang and Huan Yu contributed to acquisition of data. Chenjie Xia, Qinwen Ge, Di Chen, Peng Zhang, Zhen Zou and Peijian Tong contributed to analysis and interpretation of data. Chenjie Xia, Lvwei Xiao Peijian Tong and Shuaijie LV drafted the article or revised it critically for important intellectual content. Hongting Jin approved final version of the article to be published.

## Supporting information

Fig S1

## Data Availability

The data that support the findings of this study are available from the corresponding author upon reasonable request.
