# Supplementary figures and images for: TGF‐β/Smad2 signalling regulates enchondral bone formation of Gli1+ periosteal cells during fracture healing
Source: Cell Prolif. 2020 Sep 30;53(11):e12904. doi: 10.1111/cpr.12904 (PMC7653269; doi:10.1111/cpr.12904)

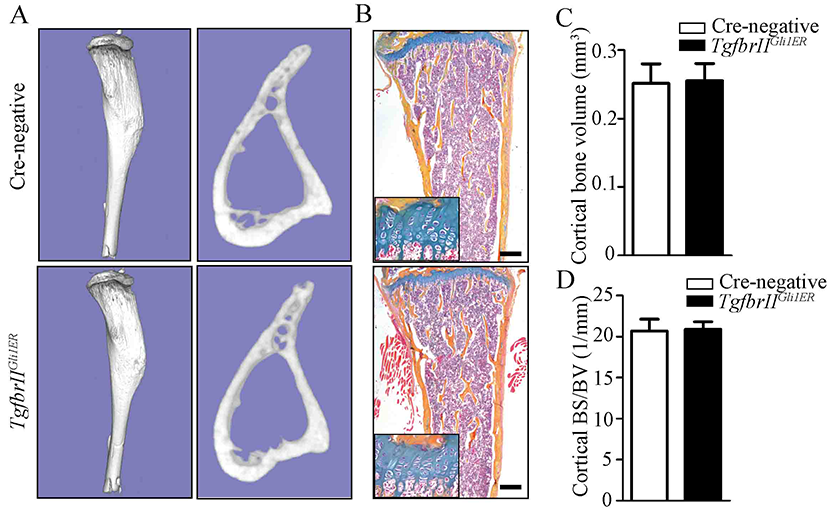

Supplement: Supplementary file 1 — Fig S1 [file CPR-53-e12904-s001.tif]
